# Temperature affects the biology of Schmidtea mediterranea

**DOI:** 10.1038/s41598-018-33355-5

**Published:** 2018-10-08

**Authors:** Nassim Hammoudi, Cédric Torre, Eric Ghigo, Michel Drancourt

**Affiliations:** 1 URMITE UMR 7278, IRD198, Institut Hospitalier Universitaire Méditerranée-Infection, Aix-Marseille Université, 19-21 Bd Jean Moulin, 13385, Marseille, Cedex 05 France; 2 Aix-Marseille University, IRD, MEPHI, IHU Méditerranée-Infection, Marseille, France

## Abstract

Studies of tissue regeneration and host-pathogen interactions using the model planarian *Schmidtea mediterranea* have been performed at an experimental temperature of 19 °C. *S. mediterranea* planarians exposed to 19 °C–32 °C were observed for survival, mobility, feeding and regeneration for three months and elimination of the *Staphylococcus aureus* pathogen over six days. *S. mediterranea* planarians died at 30 °C–32 °C after 18 days of observation but tolerated temperatures of 19 °C up to 28 °C with non-significant differences in mobility and feeding behavior. Genetic malleability tested by RNAi feeding was still efficient at 26 °C and 28 °C. Concerning the immune capacity of planarians, we reported an exacerbation of the immune response in worms infected by *S. aureus* at 26 °C and 28 °C. These observations suggest a temperature modulation of planarian stem cells and illustrate the importance of modulating experimental temperature when using planarians as model organisms to study regeneration and immune response.

## Introduction

Planarians are invertebrate flatworms belonging to the Platyhelminthe branch^1^. They are complex organisms with a central nervous system, a digestive tract and an excretory system^2^. Freshwater planarians display a unique biological capability of regeneration that has fascinated scientists since the first report by Pallas in 1766^3^. In 1898, TH Morgan described planarians as immortal animals owing to their regenerative capacity^4^. Nowadays, planarians, mainly the species *Schmidtea mediterranea*, are considered as model organisms to study the mechanisms of regeneration and development^5^. Their ability to regenerate any tissue part after amputation is due to the presence of a high proportion (25%) of stem cells called neoblasts which include pluripotent stem cells^6^. While the mechanisms regulating the development and the regeneration of planarians are widely studied, little is known about their immune capabilities. However, recent studies highlighted the resistance of planarians to infection by bacteria. Unlike other invertebrate organisms, *S. mediterranea* eliminates human pathogens such as *Staphylococcus aureus*^7^.

All these capacities make the planarian a “one of a kind” model organism to identify and study new molecules and new mechanisms of defense that could be conserved during evolution.

All studies regarding host-pathogen relationships have been carried out by incubating the planarian *S. mediterranea* at around 20 °C^7,8^. Water temperature is the most important abiotic environmental factor causing physiological changes in aquatic organisms^9^. A thermoregulation system has formed in organisms in the course of evolution^10^, it does not only regulate body temperature, but also modulates the activities of other physiological systems^11^. In fact, planarians are found almost ubiquitously in freshwater streams where they are living at a temperature between 19 °C and 22 °C^12^.

We therefore question whether adapting planarians to different temperatures above 19 °C would modify their physiology and immunological responses or not. We studied the main physiological parameters (viability, ability to feed, mobility, regeneration, genetic silencing and antimicrobial response) and determined changes in the planarian host-pathogen interactions by using *S. aureus* as an example.

## Results

### Acclimatization of planarians at temperatures >19 °C

When planarians were exposed to a cyclical rhythm of 24/48 hour reaching a maximum temperature of 30 °C, all planarians died after 24 hours of incubation whereas negative control planarians maintained at 19 °C were all alive. When planarians were exposed to temperature increased by 1 °C every 25 min up to 37 °C, all planarians exposed to ≥34 °C were found dead after 24 hours of incubation. Thus, to study the viability of *S. mediterranea* at different temperatures ranging from 19 °C to 32 °C a total of 40 planarians equally distributed into four groups were stepwise (1 °C every 25 min.) adapted and maintained at different temperatures of 26 °C, 28 °C, 30 °C and 32 °C.

### Planarian survival

**Figure 1:**
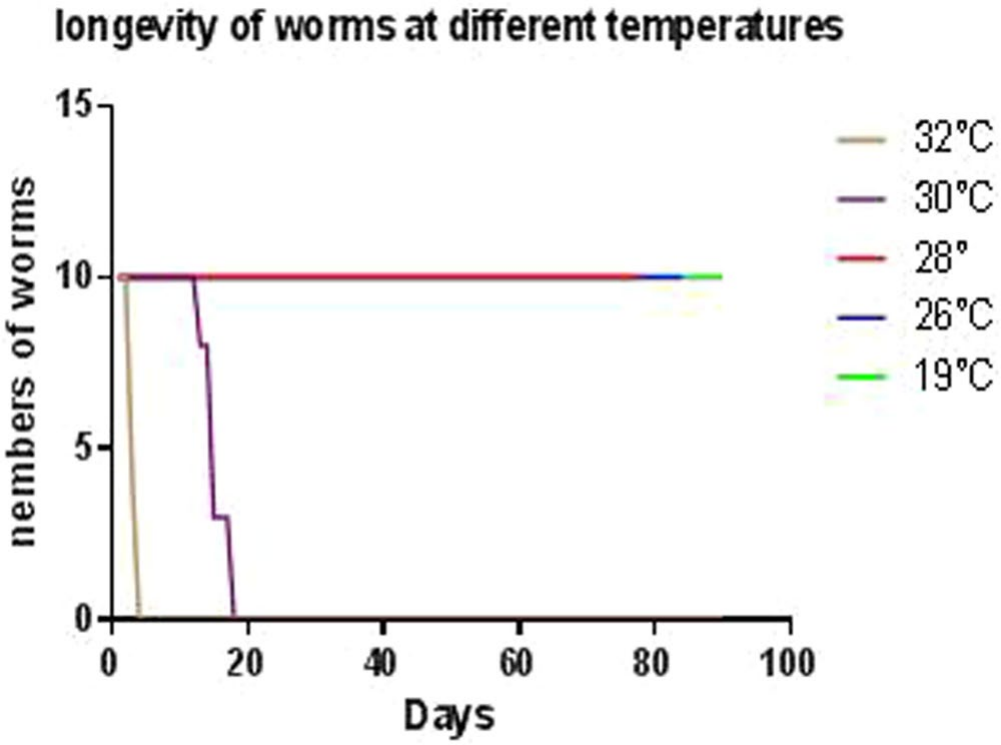
Longevity of the planarians incubated at different temperatures. The planarians exposed to a temperature increase of 1 °C every 25 min, a daily monitoring of their survival allowed us to observe the death of the worms incubated at 32 °C and 30 °C after 4 and 18 days respectively, the planarians incubated at 26 °C and 28 °C remain alive after three months of incubation.

When exposed to 32 °C, mortality was of 0% after 48 hours of incubation, 60% after 72 hours of incubation and 100% after 96 hours of incubation. When exposed to 30 °C, the mortality was of 0% after 12 days of incubation, 20% after 13 days of incubation, 70% after 15 days of incubation and 100% after 18 days of incubation. No mortality was observed when planarians were exposed to 26 °C and 28 °C for up to 3 months. This first experiment led us to conclude that *S. mediterranea* can adapt to temperatures ranging from 19 °C to 28 °C. Thus, all of the subsequent experiments were carried out at 19 °C, 26 °C and 28 °C after a one-week adaptation delay (Fig. 1).

### Spontaneous amputation of planarians at high temperature

**Figure 2:**
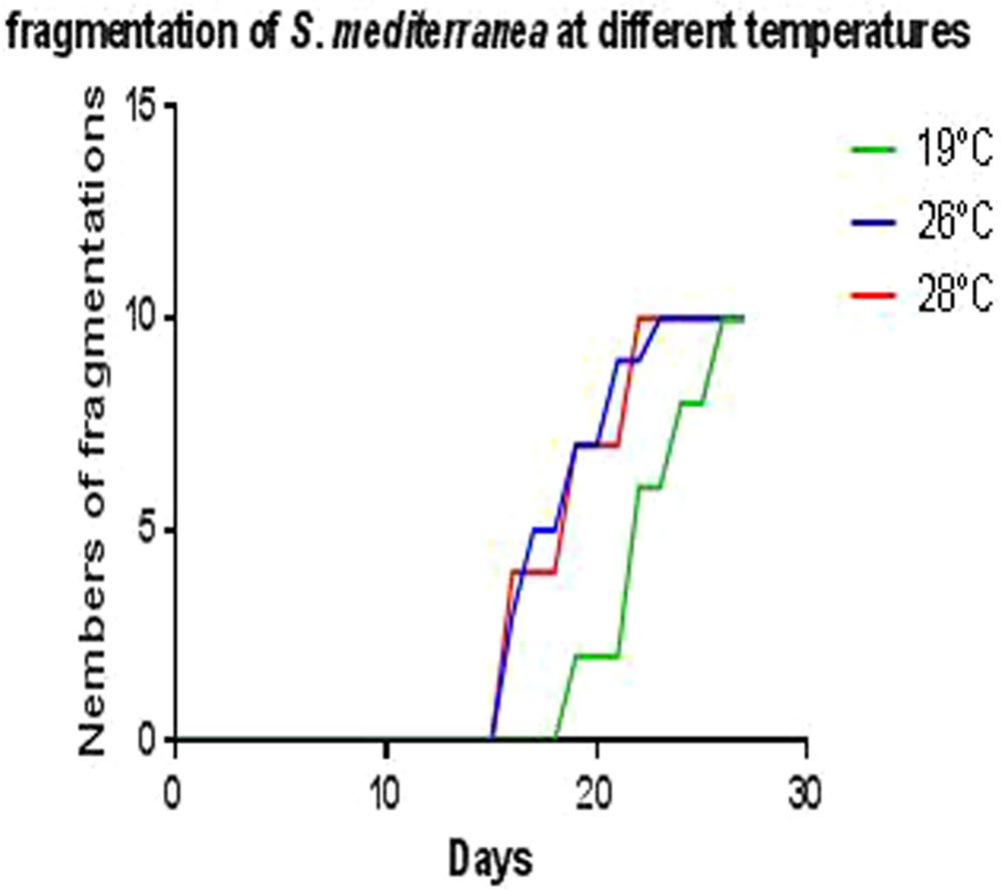
Spontaneous fragmentation of planarians incubated at different temperature. Follow-up of the number of spontaneous fragmentations observed in planarians incubated at different temperatures reveals a beginning of fragmentation from the 16th day for the worms incubated at 26 °C and 28 °C, this is observed on the 19th day at 19 °C. A doubling of the number of worms is observed after 23 days at 26 °C and 28 °C and it was not until the 26th day that it was reported at 19 °C.

When exposed at 19 °C, planarians began to cut themselves into small worms at day 19, then 50% (5/10) of the worms cut themselves at day 22 and finally 100% (10/10) cut themselves after 26 days of incubation. Nevertheless, spontaneous amputation began at day 16 for planarians incubated at 26 °C and 28 °C and our observations reported that 50% of planarians cut themselves at day 17, to reach 100% at day 22. These results enabled us to conclude that *S. mediterranea* multiplied significantly more rapidly at 26 °C and 28 °C than at 19 °C (*p* = 0.04) (Fig. 2).

### Mobility of planarians at high temperatures

**Figure 3:**
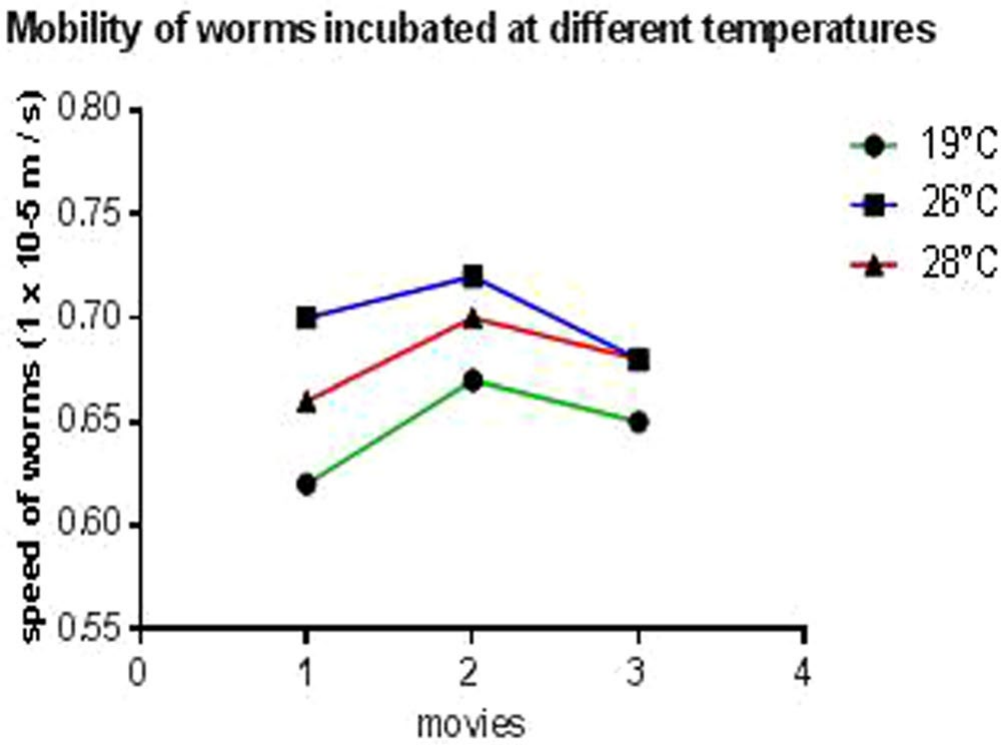
Mobility of the planarians incubated at different temperature. The biostatistical analysis with the BiostaTGV software of the results obtained from the videos of mobility of the worms allowed us to calculate the average speeds of movements in each realized film. This revealed to us 0.65 × 10^−5^ m/s, 0.7 × 10^−5^ m/s, and 0.68 × 10^−5^ ± 0.02 m/s (*p* = 0.06) at 19 °C, 26 °C, 28 °C respectively.

The average speed of the worms calculated on the three films was of 65 × 10^−5^ m/s at 19 °C, 70 × 10^−5^ m/s and 68 × 10^−5^ m/s at 26 °C and 28 °C, respectively. This implies that the worms incubated in these temperatures showed a non-significant difference (*p* = 0.06) in the speed of their displacement (Fig. 3, Supplementary Video).

### Feeding behavior of planarians at high temperatures

**Figure 4:**
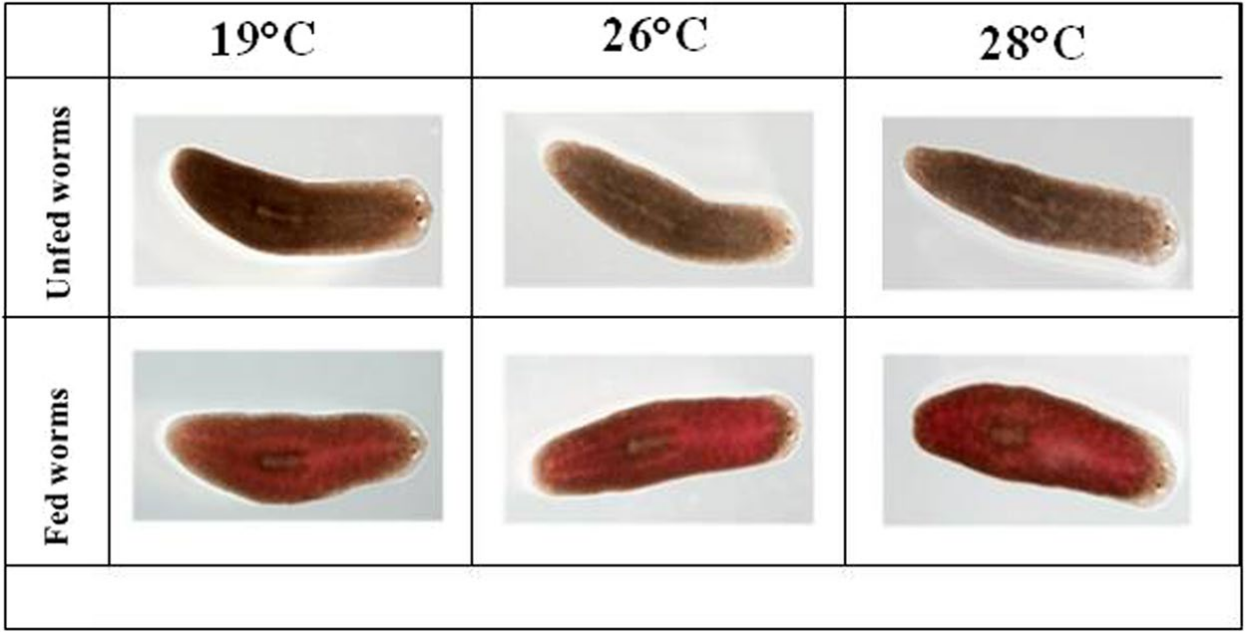
Presentation of phenotypes of fed planarians and incubated at different temperatures, namely 19, 26, 28 °C.

**Figure 5:**
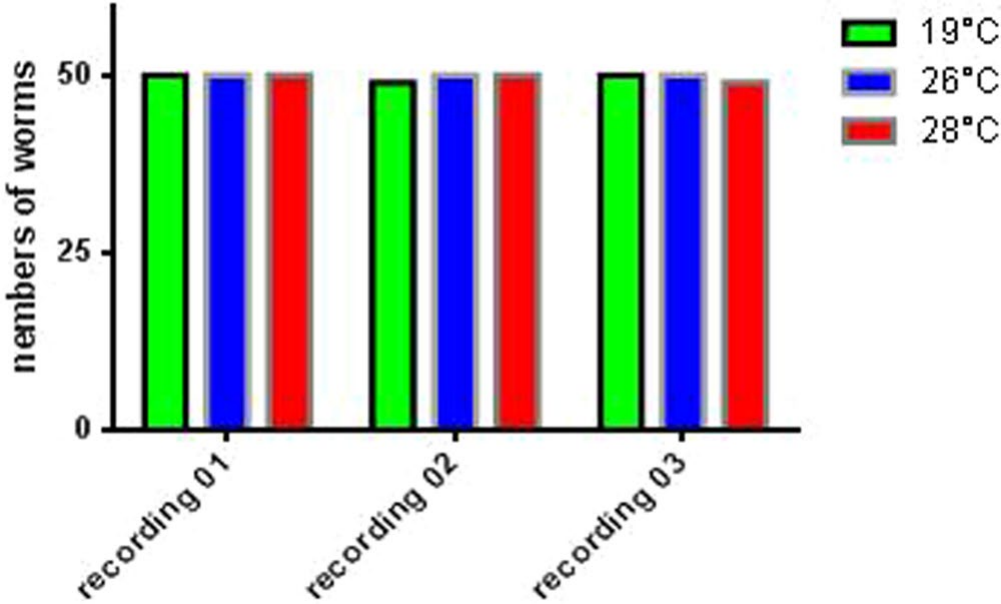
Feeding behavior of the planarians incubated at different temperature. Three planarian groups (n = 50) adapted to 19, 26, 28 °C, are fed three consecutive times at an interval of 2 days to estimate their aptitude to eat, at 19 °C there were 50, 49, 50 ± 0.5773 fed, at 26 °C 50 worms fed in all experiments, finally, at 28 °C, 50, 50, 49 ± 0.5773 worms fed.

**Figure 6:**
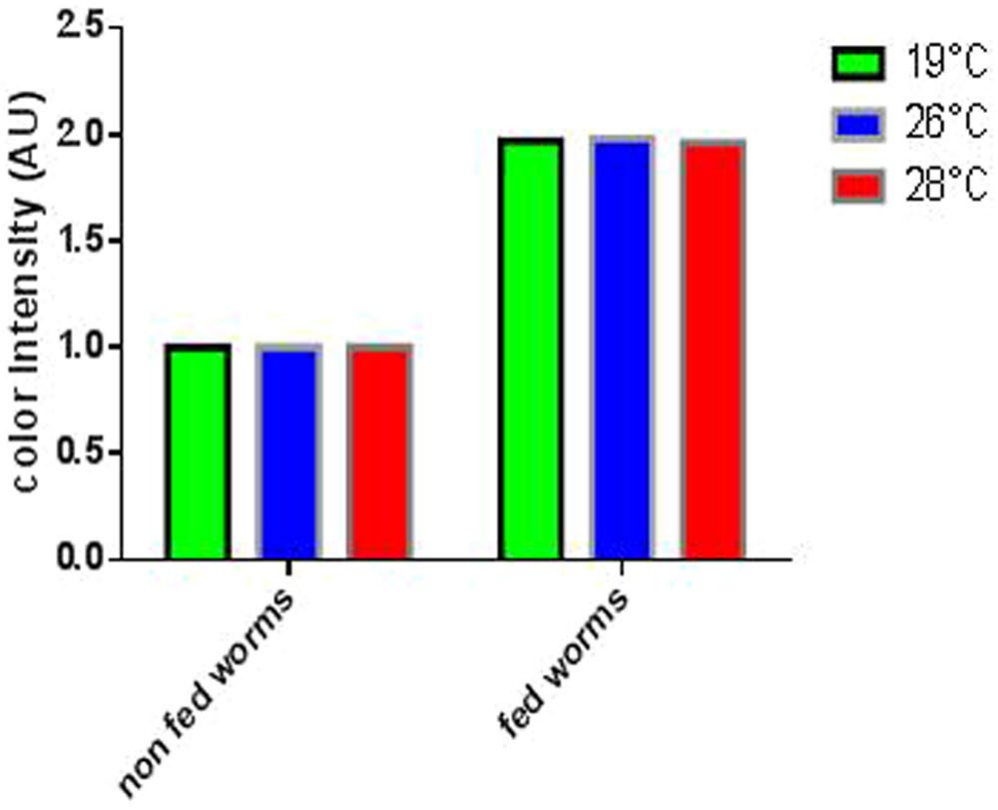
Intensity of coloration after the feeding of the planarians incubated at different temperatures. The feeding of the worms is followed by a calculation of the color intensity of the worms using the ImageJ software, this analysis shows us a non-significant difference in the color intensity of the worms incubated in different temperatures. Indeed, 1.97, 1.98, 1.96 (UAC) were obtained at 19, 26, 28 °C respectively.

In order to define the effect of temperature above 19 °C on the ability of planarians to feed, three feedings were carried out for three groups of planarians (n = 50) incubated at different temperatures (19 °C, 26 °C and 28 °C) (Fig. 4). The results showed a non-significant difference in the number of planarians fed depending on the temperature. When exposed to 19 °C, 99% of planarians were red-colored after food intake; 100% when exposed at 26 °C and 99% when exposed at 28 °C. There was obviously no significant difference between the feedings at different temperatures *(p* = 0.6) (Fig. 5). The color intensity of planarians was 1.97, 1.96 and 1.98 AU (arbitrary unit) at 19 °C, 26 °C, and 28 °C respectively. We thus concluded that the feeding of *S. mediterranea* in the present study was not affected by temperature in the 19 °C–28 °C interval (Fig. 6).

### Regeneration of planarians at high temperatures

**Figure 7:**
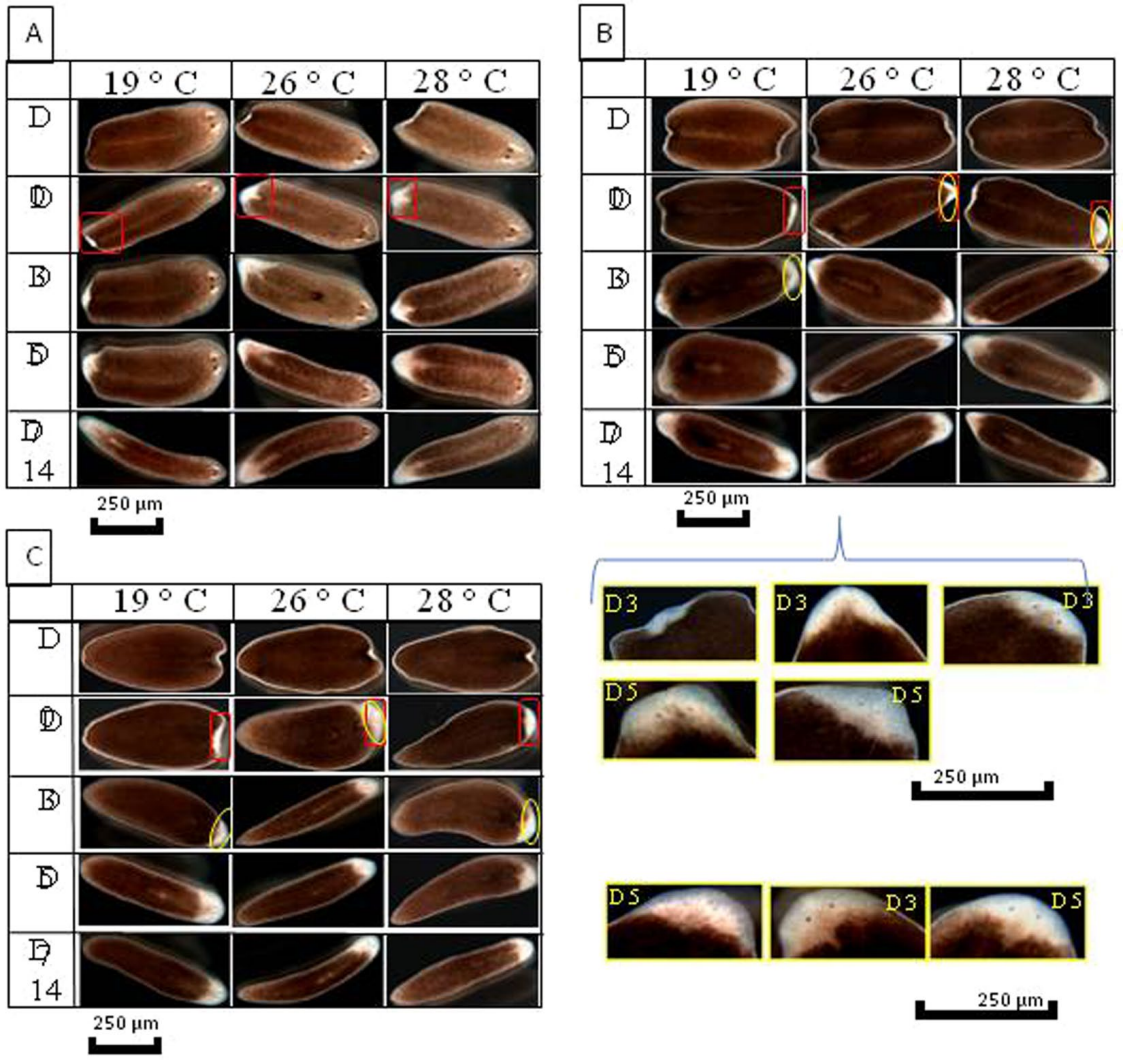
Regeneration of planarians after amputation of the head and tail. (**A**) Regeneration of the head: appearance of the blastema at Day 3 on all the fragments, the blastema formation is complete after Day 7, and the regeneration ends at Day 14. (**B**) Regeneration of the trunk: we see the appearance of the blastema on Day 3 with the formation of the eyes on the worms incubated at 26 °C, concerning the worms incubated at 19 and 28 °C the eyes are formed on Day 5. (**C**) Regeneration of the trunk: at Day 3 post-amputation, we see the formation of blastema on all the fragments at different temperatures, this is followed by the appearance of the eyes in worms incubated at 26 °C; at 19 °C and 28 °C, the eyes only appear at Day 5. The complete formation of the blastema is made on Day 7 and the completion of the regeneration process is observed on Day 14.

**Figure 8:**
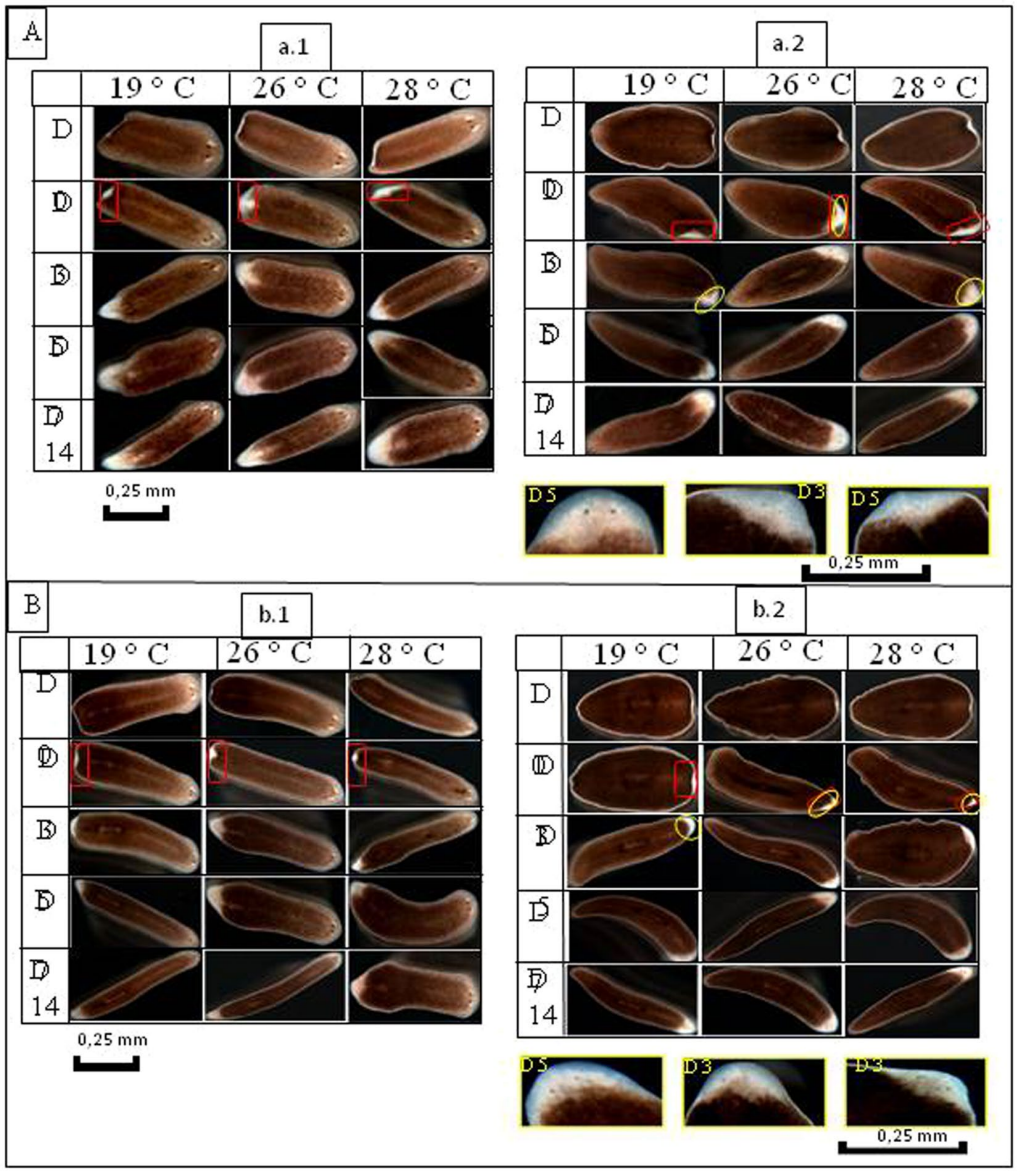
(**A**) Regeneration of planarians after amputation of the head. (a.1) Regeneration of the head: appearance of the blastema on all the fragments on Day 3, then completely formed on Day 7, and the regeneration ends on Day 14. (a.2) Regeneration of the trunk-tail: formation of the regeneration blastema on Day 3 and an appearance of the eyes in worms incubated at 26 °C is reported, however, at 19 °C and 28 °C the eyes do not appear until Day 5. (**B**) Regeneration of planarians after amputation of the tail. (b.2) Regeneration of the head-trunk: after the section of the tail, the formation of blastema begins on Day 3 and ends on Day 7 for all the fragments, and complete worms are formed on Day 14. (b.2) Regeneration of the tail: characterized by the appearance of the eyes on blastema of worms incubated at 26 and 28 °C, this observation is reported on Day 5 for worms incubated at 19 °C.

A daily monitoring was carried-out during 14 days on the two-pieces (head-tail) and three-pieces (head-trunk-tail) amputated worms. Irrespective of the incubation temperature and whatever the part of the worm, we observed the regeneration blastema’s appearance on all fragment types after three days post-amputation independently of the temperature (Figs 7 and 8). Moreover, in the group of worms that were cut into three pieces, complete body regeneration from the head was observed at seven days post-amputation at different temperatures. At 19 °C, complete regeneration of the head and tail from the trunk was observed at seven days post-amputation. However, this same observation was made at five days post-amputation for the same worm parts incubated at 26 °C and 28 °C. At 19 °C, the appearance of the eyes from the trunk was confirmed at five days post-amputation. On the other hand, at 26 °C and 28 °C, this observation was made at three days post-amputation.

Concerning the tail fragments, the appearance of the eyes was confirmed at three days post-amputation for worms incubated at 26 °C. Moreover, at 19 °C and 28 °C, it is only at five days post-amputation that the eyes were formed (Fig. 7C). For all temperatures, the regeneration of worms cut into two pieces (head; trunk + tail) was completed at seven, five and seven days post-amputation at 19 °C, 26 °C and 28 °C, respectively (Fig. 8A). The appearance of the eyes on the tail fragments was confirmed at five days post-amputation at 19 °C and 28 °C. Besides, at 26 °C, the regeneration of the eyes was observed at three days post-amputation (Fig. 8a.2).

**Figure 9:**
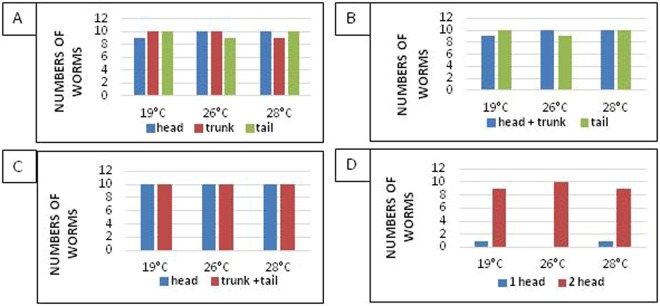
Quantification of the different phenotypes observed after fragmentation. (**A**) Number of worms regenerated after three cuts (head, trunk, tail) with a P = 1 according to temperature. (**B**) Number of worms regenerated after two cuts (head + trunk, tail) P = 0.64 according to temperature. (**C**) Number of worms regenerated after two cuts (head, trunk + tail) P = 0.46. (**D**) Number of worms with one or two heads after invalidation of β-catenin gene as a function of temperature *P* = *1*.

Finally, for the worms cut into two pieces (head + trunk; tail), the complete regeneration of the fragments was completed at seven days post-amputation at 19 °C. On the contrary, this was observed at five days post-amputation for the fragments incubated at 26 °C and 28 °C (Fig. 8B). At 19 °C, we noticed the appearance of the eyes at five days post-amputation from the tail, this phenomenon was observed at three days post-amputation for worms incubated at 26 °C and 28 °C (Fig. 8b.2). The quantification of all phenotypes observed in these experiments is presented in the Fig. 9. In conclusion, the regeneration of the amputated worms kept at 26 °C and 28 °C was slightly faster than the regeneration of those kept at 19 °C.

### RNA interference invalidation of planarians

**Figure 10:**
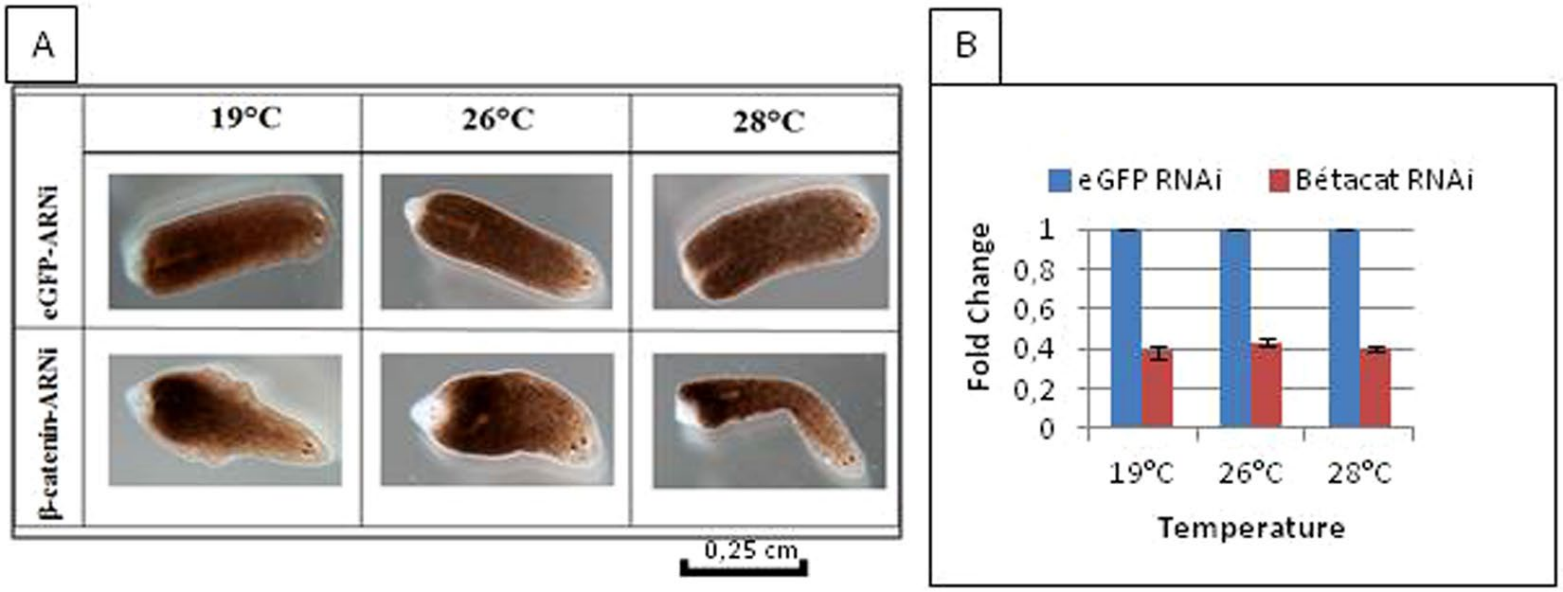
Regeneration of the planarian head-trunk part invalidated for the *eGFP* or *β-catenin* genes. (**A**) After the genes were invalidated, the worms were cut. At day 5 of the regeneration, there was appearance of the head instead of the tail in worms invalidated for the β-catenin gene, a normal regeneration in worms invalidated for the gene eGFP (jellyfish gene non-existent in the Planarian genome). (**B**) The fold change of the β-catenin and eGFP genes invalidated in 10 planarians incubated in the three different temperatures (19 °C, 26 °C, 28 °C) reveals a non-significant difference with P = 0.99.

In planarians for which the eGFP gene (negative control) and the β-catenin gene were inactivated, we followed the evolution of the regeneration process from incubated fragments (head-trunk) at different temperatures. The results showed the formation of regenerative blastema on all fragments after the third day post-amputation. This blastema formation was followed by a differentiation characterized by the appearance of the eyes at the posterior site in the β-catenin invalidated planarians. On the contrary, in the group of control worms, we noticed a normal regeneration (Fig. 10A) which quantification is provided in Fig. 9D. Invalidation of the eGFP and β-catenin genes as measured by RT-PCR, yielded no significant difference with incubation temperature (P = 0.99) (Fig. 10B). These observations were reproducible at all incubation temperatures. The phenotype of the β-catenin invalidation appeared in all incubation temperatures, suggesting that RNA interference is possible on worms incubated at high temperatures.

### Infection of planarians with *S. aureus*

**Figure 11:**
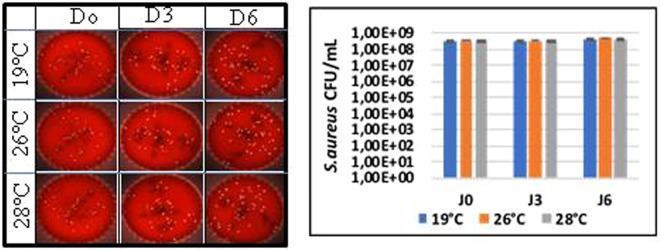
Test viability of *S. aureus* incubated in water at different temperatures. After incubation of the bacteria in water at 19 °C, 26 °C and 28 °C the bacteria are enumerated every 3 days by counting the CFUs. Results showed a non-significant difference as a function of the incubation temperature with P = 0.99.

**Figure 12:**
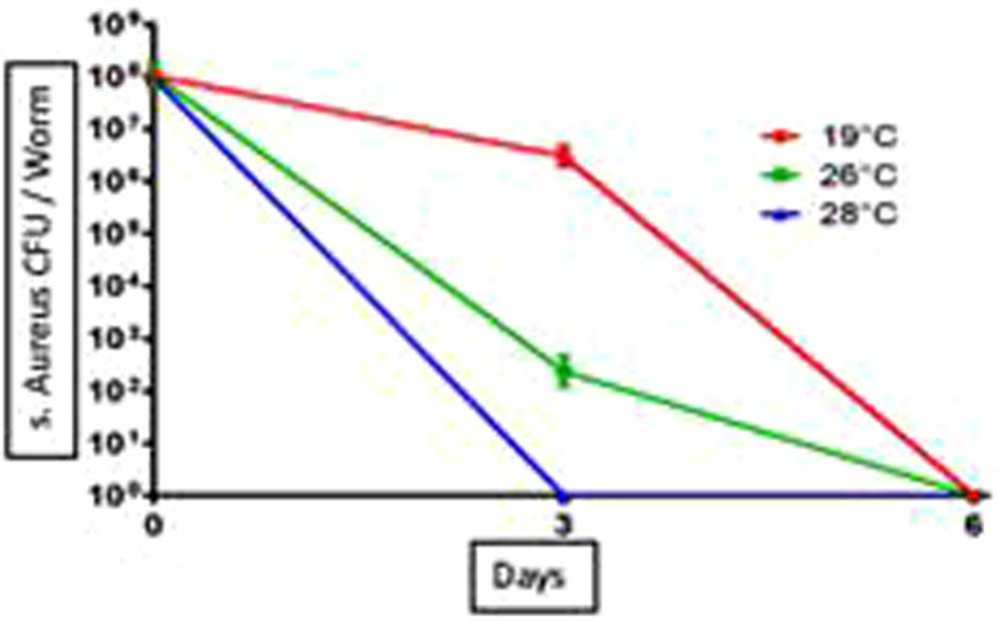
Kinetics of elimination of *S. aureus* by planarians. After infection of the planarians with *S. aureus*, the bacteria were enumerated every 3 days by counting the CFUs. The elimination kinetics revealed a clearance at Day 6 post-infection at 19 °C and 26 °C, however this clearance was observed on Day 3 in worms incubated at 28 °C. Elimination of bacteria by planarians was faster at 28 °C and 26 °C compared to control 19 °C. The results are expressed as mean ± standard deviation (10 worms per point, n = 3, *p < 0.05). The results are analyzed with GraphPad Prism© software using the non-parametric Mann-Whitney U test.

**Figure 13:**
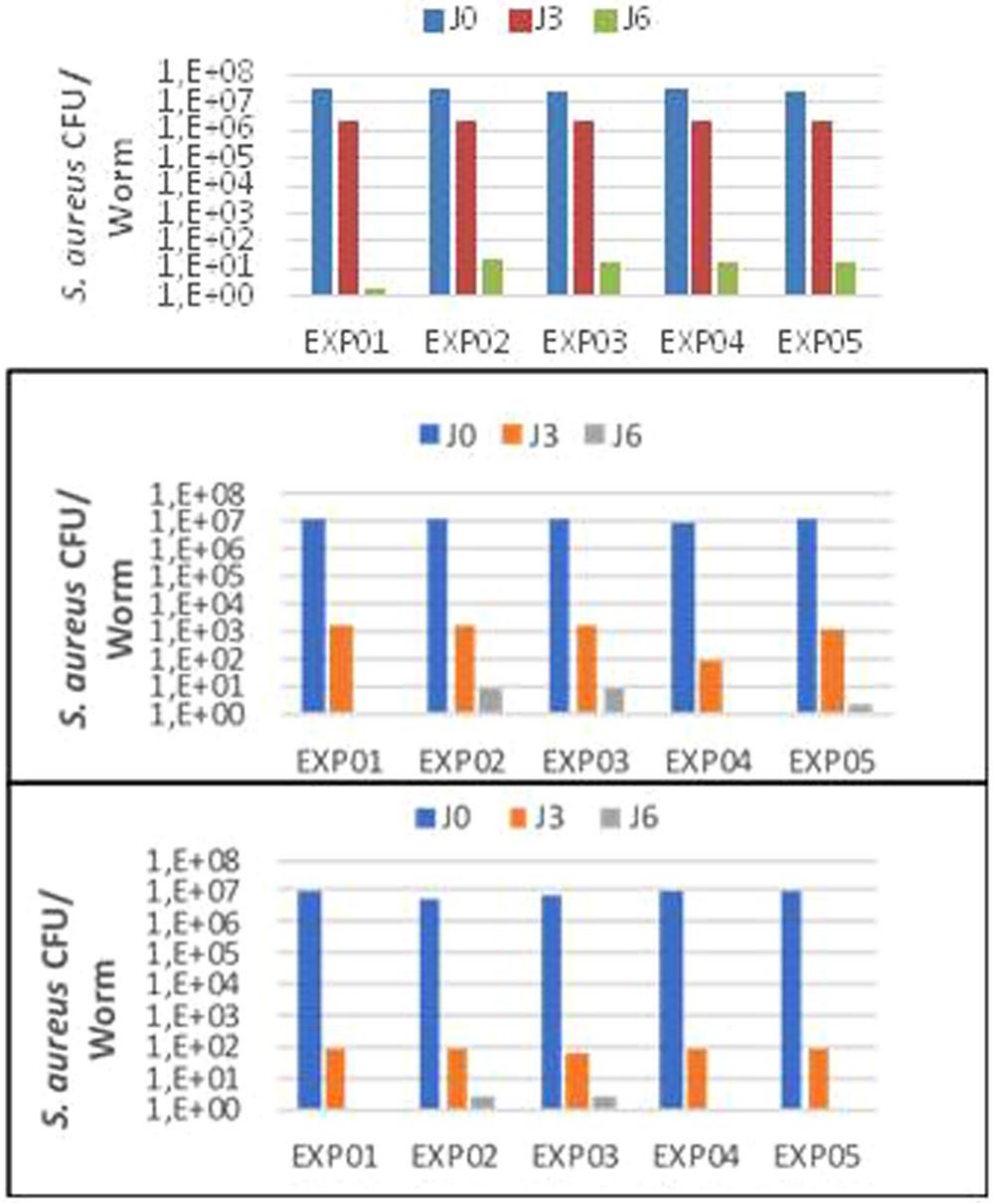
Reproducibility test of the results of the elimination of *S. aureus* by the planarians. (**A**–**C**) Represent five replicates of *S. aureus* eliminated by the planarians in each incubation condition 19 °C, 26 °C and 28 °C, showing non-significant differences with P = 0.89; 0.58; 0.99, respectively.

Finally, we assessed the effect of the change in temperature on the ability of planarians to eliminate bacteria. We first ensured that exposing *S. aureus* bacteria to the various temperatures did not significantly alter their viability (Fig. 11). Then, *S. mediterranea* planarians adapted to temperatures of 19 °C, 26 °C and 28 °C were infected with 10^9^ CFU of *S. aureus* for three hours (considered as time 0). The fate of the bacteria was then monitored for six days by determining the number of CFUs. In the control group of planarians exposed to 19 °C, *S. aureus* was eliminated in six days as previously reported^7^. When planarians were exposed to 26 °C, the number of *S. aureus* bacteria was of 10^2^ CFUs after three days of infection compared to 10^4^ CFUs in the control group (19 °C). When planarians were exposed to 28 °C, the bacteria were completely eliminated after three days (Fig. 12). The experiment was repeated five times and yielded reproducible results (Fig. 13). Taken together, these results suggest that planarians have exacerbated antibacterial capabilities for temperatures above 19 °C in the 19 °C–28 °C interval.

## Discussion

Among all environmental parameters, temperature is one of the most important fundamental factor^9^ playing an important role in the metabolism of organisms^13^. Furthermore, it has been reported in several studies that the activities of the different physiological systems are modulated by the thermoregulation system^13–15^ which is involved in the development of many organs^10^. In 1897, Lillie *et al*. reported an inversely proportional relationship between the incubation temperature and the time required to reach a given morphological stage in *Rana pipiens*^16^. The development of an immune response is often associated with fever^17^. Recently, Kim *et al*. (2017) reported an increase in the immune response of “sand-fish” at high temperature of culture^18^.

Planarians are aquatic flatworms living in fresh waters at 19 °C to 22 °C. They are endowed with an extraordinary capacity of regeneration and an impressive ability to eliminate human pathogens^4,7,19–22^. Planarians are always confronted to temperature variations in their natural environment, which is why in this study we used the *S. mediterranea* planarian species to establish a model that allowed the study of microbial infections at different temperatures.

We have exposed the planarians to temperatures over 19 °C. At first, we increased the temperature by 1 °C every 25 min. Following this method, we were able to heat up the worms up to 37 °C. Nevertheless, after 24 hours, we found that all the worms incubated above 34 °C died. Secondly, the aim was to know if the rhythm of acclimation of this species of planarians would have an effect on their tolerance to temperature. To do this, we carried out a second adaptation method where we increased the temperature by 1 °C applying a 24/48-hour cyclic rhythm. This method allowed us to incubate them in extreme temperatures of exactly 30 °C, which caused the death of all planarians 24 hours later. This enabled us to choose the first method (increase of the temperature by 1 °C every 25 min) for the rest of the work. Following this method, we determined a range of temperatures from 19 °C to 28 °C in which planarians remained alive for more than three months. It should be noted that the planarians incubated in temperatures above 28 °C died gradually during the 18 days of incubation. In determining the temperature range for which the planarians adapted, we investigated whether their ability to feed was affected by the change in temperature.

It is a question of importance, since feeding of planarians is used to induce genetic invalidation and to infect them with bacteria^7^. Thus, we observed that planarians keep their ability to feed during incubation at 26 °C and 28 °C compared to what we observed at the usual incubation temperature of 19 °C. This observation is in contradiction with the study by Kim *et al*., which reports that an increase in the temperature of the culture water affects negatively the feeding behavior of *Anoplopoma* fimbria^23^. We were interested in the mobility of planarians at different temperatures, so we estimated the speed of their displacement at 19 °C, 26 °C and 28 °C. Despite the fact that we observed 10 individual worms over three replicate experiments, the results we obtained did not yield significant difference using the standard 0.05 P value. Nevertheless, these observations did not eliminate the possibility that in nature, over a larger population of worms, temperature variation may somewhat alter the motility of a subpopulation of the worms. Indeed, a previous study suggested that an increase in temperature increases the locomotion of the nematode *C. elegans*^23^. This particular point may warrant further studies.

The ability of *S. mediterranea* to regenerate is well-studied, but only in their usual incubation temperature (19 °C)^24,25^. Here, we were interested in the study of this ability under the new thermal conditions described here. As reported by Morgan in 1898, the planarians regenerate completely in two weeks at 19 °C^4^. The results of the regeneration process follow-up of the planarians incubated at 26 °C and 28 °C allowed us to note a complete regeneration of the different parts after 14 days. In addition, the change in incubation temperature induced a faster regeneration of the head from the trunk and tail incubated at 26 °C and 28 °C compared to the fragments incubated at 19 °C. This fast regeneration of the head was visible thought an early development of the eyes on the fragments incubated at 26 °C and 28 °C. Thus, the increase of the incubation temperature accelerated the development of organs in the planarians. Our observation agrees with the results reported by Lillie *et al*., in the embryogenesis of *R. pipiens*^16^.

Before the molecular era, the generation of two-headed planarians was possible. Morgan and Child observed that when a planarian is cut into extremely thin fragments, two-headed planarians often develop^4,26^. In 2012, Almuedo-Castillo *et al*. reported the effect of β-catenin invalidation on planarian regeneration. The β-catenin invalidation lead to the loss of antero-posterior patterning in regeneration inducing a double-head phenotype^27^. Although other organisms such as *C. elegans* and vertebrates also have β-catenin genomic duplicates, planarians are the only organisms known to present a clear functional specialization between different paralogues^27^. Thus, we observed the regeneration of a second head at the posterior site, not only on the planarians incubated at 19 °C, but also on those acclimated at 26 °C and 28 °C. These results enabled us to use the genetic invalidation tool on planarians incubated at high temperatures, since it did not alter their genetic malleability.

During this study, we determined the effect of temperature on the physiology and the ability of *S. mediterranea* to eliminate bacterial pathogens for humans, here using *S. aureus*. Monitoring the kinetics of elimination of this bacterium showed the conservation of the capacity of the planarians to eliminate *S. aureus* in the different thermal conditions tested. Our results are consistent with those reported by Abnave *et al*.^7^. By comparing the reduction in the number of CFUs found on the third day of infection, we observed a reduction by about 10^5^ in the planarians incubated at 26 °C. The complete elimination of this bacterium by planarians incubated at 28 °C was faster, with less CFU on day 3 after infection compared to those incubated at 26 °C. This suggests that the incubation temperature exacerbates the planarians’ ability to eliminate pathogens. This is consistent with the observations reported by Kalenova and Fisher (2005) about the relationship between the thermoregulation system and the immune system^17^. Besides, there is a correlation between the increase of regeneration capacity and the increase of antibacterial immunity at high temperature, suggesting an involvement of planarian stem cells in the immune process, as previously shown in the context of immune memory (Torre *et al*.)^21^.

In conclusion, during this study, we set up a new study model using the *S. mediterranea* planarian which is adapted to various incubation temperatures, allowing us to carry out experiments in order to understand the impact of thermal changes on host-pathogen interactions. This is particularly important when it comes to pathogens requiring specific incubation temperatures. Further studies could focus on the signaling pathways leading to their elimination in these conditions. In addition, the acceleration of regeneration observed at 26 °C opens up prospects for work on tissue regeneration.

## Materials and Methods

### Planarians and bacteria

The planarians used in this work belong to the species *S. mediterranea* (asexual clonal line ClW4). Planarians were maintained in filtered tap water (Ultracarb Doulton Newcastle-under-Lyme, England), and autoclaved tap-water at 19 °C on 0.2-µm pore size Whatman membrane filters nylon (Nalgene, Thermo Scientific, Waltham, US) and fed once per week with grinded calf liver. Planarians were starved for at least one week and matched by size prior to the experiments. *S. aureus* ATCC25923 strain was grown on blood agar plates (bioMérieux, La Balme-les-Grottes, France) at 37 °C under 5% CO_2_ atmosphere. Its identity was confirmed by matrix-assisted laser desorption ionization time-of-flight mass spectrometry (MALDI-TOF-MS) biotyping^28^. The viability of *S. aureus* bacteria in water at 19 °C, 26 °C and 28 °C was determined by plating an inoculum of 10^9^ CFUs previously exposed to 19 °C, 26 °C or 28 °C on blood-agar plates (bioMérieux) at 37 °C under 5% CO_2_ atmosphere for 48 hours. Colonies were counted in order to determine the survival of *S. aureus*. This experiment was done in triplicate.

### Temperature adaptation

Two methods were developed to adapt planarians at temperatures >19 °C. The first method was adapted from Russier-Delolme (1972) and consisted in keeping planarians in an incubator at 19 °C and increasing the temperature with a rate of 1 °C every 25 min^29^. The second method consisted in keeping planarians in an incubator at 19 °C and increasing the temperature with a rate of 1 °C every 24 h or 48 h. For this, 120 *S. mediterranea* worms grown at 19 °C, were selected according to their size (about 1 cm) and were randomly grouped into two groups of 60 worms, one group per method. After validating the most efficient acclimation protocol, 40 planarians divided into four subgroups of 10 worms each were adapted and maintained at different temperatures (26 °C, 28 °C, 30 °C and 32 °C) to study longevity and monitor multiplication.

### Planarian behavior

**Figure 14:**
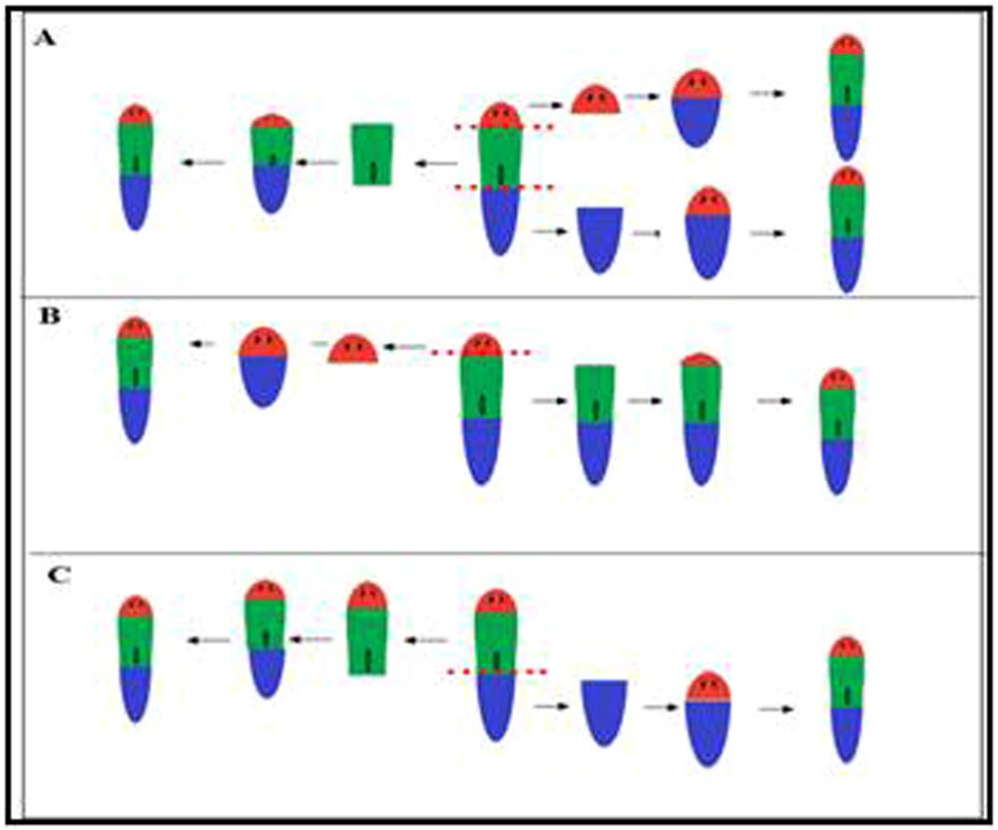
Fragmentation of planarians. (**A**) Fragmentation of the planarians in three parts head, trunk, tail. (**B**) Amputation of the head. (**C**) Amputation of the tail (ref.^4^).

The survival, mobility, feeding and regeneration were measured for planarians kept at every temperature. In every experiment, negative control consisted in worms kept at 19 °C. To measure the mobility, the speed of 10 planarians was measured from 1.30 min videos made on millimeter paper using a camera (Canon EOS 100D, Taiwan, Japan). The average speed and its standard deviation were calculated after analyzing the videos using the BiostaTGV software (https://marne.u707.jussieu.fr/biostatgv/). The experiment was done three times. To measure the feeding, planarians were fed with grinded calf liver containing 0.6% E127 red food dye (McCormick, Avignon, France). Pictures were taken three hours after the feeding. The intensity of red dye was measured using ImageJ software (National Institutes of Health). To measure regeneration, three groups of planarians were observed: in group (1), 10 planarians had their head, trunk and tail amputated. In group (2), 10 planarians had their (head, trunk) and tail amputated; in group (3), 10 planarians had their head and (trunk and tail) amputated (Fig. 14). The regeneration process was followed during 14 days through picture capture. All pictures were taken using a Leica M135FC (Mannheim, Germany) with a camera from DMK, Imaging Source (The Imaging Source Europe GmbH, Bremen, Germany) and pictures were analyzed using the software IC Captur 2.4 (The Imaging Source Europe GmbH).

### Planarian feeding with *S. aureus*

The planarians were fed *S. aureus* using a protocol adapted from a dsRNA feeding method (Reddien *et al*., 2005) as previously described^7^, with a few modifications. Briefly, bacterial pellets containing 1.10^9^ colony-forming units (CFU)/mL were suspended into 50 µL of homogenized liver, mixed with 20 µL of 2% ultra-low-gelling-temperature agar (Sigma-Aldrich, Darmstadt, Germany) and 0.3 µL of E127 red food dye and the mixture was allowed to solidify on ice. Room temperature solidified food was fed to the planarians. After feeding for three hours (defined as day 0), the planarians were extensively washed with tap water and used for experiments. The experiment was repeated five times.

### CFU Counting

Planarians were collected and homogenized in 100 µL of sterile phosphate buffered saline (PBS) (Life Technologies Corporation, Grand Island, USA). The lysate was passed five times through a sterile syringe with a 29G needle to disrupt planarian tissue clumps. 10 µL of ten-fold serial dilutions in one mL of PBS were plated onto 5% sheep-blood agar plates (bioMérieux) and incubated at 37 °C under a 5% CO_2_ atmosphere for 24 h. CFUs were then counted using an automatic Scan 1200 scanner (Interscience international, Saint Nom la Bretèche, France).

### Delivery of dsRNAs via feeding

dsRNAs were delivered as previously described^12^. Briefly, worms were submitted to three rounds of RNAi feeding (one round every three days). Three days after the last RNAi feeding, planarians were cut to assess the appearance of β-catenin-RNAi phenotype. The quality of gene down-regulation was controlled via real time-qPCR^7,30^. The primers used for RT-qPCR targeted smed-β-catenin (left primer: 5′-CCAGATACTCCTGGTAGTAC-3′; right primer: 5′-ACTCCAAGTGATTGAACAG-3′). The results of smed-β-catenin RT-PCR were normalized using the results of RT-PCR amplification of the housekeeping gene *smed-ef2*^31^.

### Statistical analysis

All results were expressed as means ± standard deviation and analyzed using the non-parametric Mann-Whitney U test using BiostaTGV software (https://marne.u707.jussieu.fr/biostatgv/). Differences were considered significant for *p* values < 0.05.

## Electronic supplementary material

Dataset 1
