# Supplementary material for: Temperature affects the biology of Schmidtea mediterranea
Source: Sci Rep. 2018 Oct 8;8:14934. doi: 10.1038/s41598-018-33355-5 (PMC6175859; doi:10.1038/s41598-018-33355-5)
Supplement: Supplementary file 1 — Dataset 1 [file 41598_2018_33355_MOESM1_ESM.pdf]

19°C	26°C	28°C
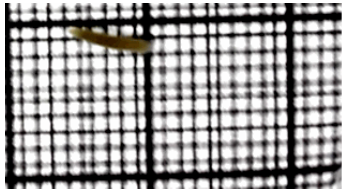	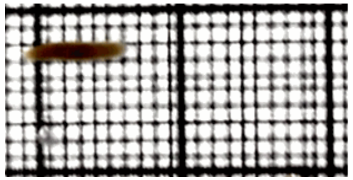	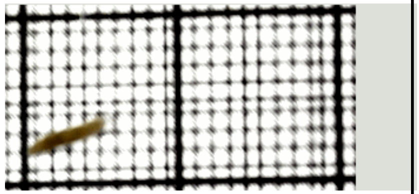
**0.65×10^{-3} m/s**	**0.70×10^{-3} m/s**	**0.68×10^{-3} m/s**

Fig 3.S: Mobility of planarians incubated at different temperatures.
Averaged speed was of $0.65 \times 10^{-5} \pm 0.02$ m/s, $0.7 \times 10^{-5} \pm 0.02$ m/s and 0.68×10^{-5} m/s $\pm$ 0.02 ($p = 0.06$) at 19°C, 26°C, 28°C respectively.
